# Impact of caveolin-1 expression on prognosis of pancreatic ductal adenocarcinoma

**DOI:** 10.1038/sj.bjc.6600619

**Published:** 2002-11-04

**Authors:** M Suzuoki, M Miyamoto, K Kato, K Hiraoka, T Oshikiri, Y Nakakubo, A Fukunaga, T Shichinohe, T Shinohara, T Itoh, S Kondo, H Katoh

**Affiliations:** Department of Surgical Oncology, Division of Cancer Medicine, Hokkaido University Graduate School of Medicine, Kita-15, Nishi-7, Kita-ku, Sapporo, Hokkaido 060-8638 Japan; Department of Pathology, Teinekeijinkai Hospital, 1-12, Maeda, Teine-ku, Sapporo, 006-0811 Japan; Department of Surgical Pathology, Hokkaido University Hospital, Kita-14, Nishi-5, Kita-ku, Sapporo, 060-8648 Japan

**Keywords:** caveolin-1, pancreatic carcinoma, immunohistochemistry, prognosis

## Abstract

Caveolin-1 is a major component of caveolae and plays a regulatory role in several signalling pathways. Caveolin-1 was recently identified as a metastasis-related gene in prostate cancer. The clinical effects of caveolin-1 expression in pancreatic carcinoma, however, remain unknown. In this study, we have investigated the relationship between caveolin-1 expression and the clinicopathologic variables and clinical outcome in 79 patients with pancreatic adenocarcinoma undergoing surgical resection. Caveolin-1 expression was determined by immunohistochemistry, using a polyclonal anti-caveolin-1 antibody. Patients were divided into two groups based on the extent of caveolin-1 expression: a negative expression group (immunoreactivity in less than 50% of cells) and a positive expression group. Positive caveolin-1 immunostaining was detected in 32 cases (40.5% of total), while non-neoplastic ductal epithelium showed little or no staining. Positive caveolin-1 expression was correlated with tumour diameter (*P*=0.0079), histopathologic grade (*P*=0.0272) and poor prognosis (*P*=0.0008). Upon multivariate analysis with Cox's proportional hazards model, positive caveolin-1 expression was shown to be an independent negative predictor for survival (*P*=0.0358). These results suggest that caveolin-1 overexpression is associated with tumour progression, thereby indicating a poor prognosis for certain patients undergoing surgical resection for pancreatic carcinoma.

*British Journal of Cancer* (2002) **87**, 1140–1144. doi:10.1038/sj.bjc.6600619 www.bjcancer.com

© 2002 Cancer Research UK

## 

Pancreatic carcinoma is one of the most common and lethal malignancies (Niederhuber *et al*, 1995). Despite developments in surgical and non-surgical therapies, significant improvements in long-term survival have not been realized. The majority of patients present an advanced disease at the time of diagnosis, because of the tumour's characteristic rapid progression and late detectability. Accordingly, the resectability rate for pancreatic carcinoma is low in most series. Tumour resection is performed in only 9–36% of all patients. Even in those patients fortunate enough to have a resectable lesion, the 5-year survival rate following resection is 11–24% (Conlon *et al*, 1996; Yamamoto *et al*, 1998; Sener *et al*, 1999). The reasons behind the aggressiveness of pancreatic carcinoma are not clearly understood. Therefore, the identification of biological markers that correlate with clinicopathologic variables or prognosis is important in understanding the characteristics of this neoplasm and selecting patients who would benefit most from multimodality treatment.

The 21–24 kDa molecule, caveolin-1, is a major structural component of caveolae, 50–100 nm vesicular invaginations of the plasma membrane that mediate molecular transport and signal transduction activities. Caveolin-1 plays a regulatory role in several signalling pathways, including the Src family tyrosine kinase, epidermal growth factor receptor, Neu/HER2 (c-*erb*B2), protein kinase C, transforming growth factor (TGF)-β/SMAD, and Wnt/beta-catenin/lef-1 pathways (Engelman *et al*, 1998a; Galbiati *et al*, 2000; Razani *et al*, 2001). In prostate cancer, caveolin-1 has been found to be a metastasis-related gene with an independent prognostic value for patients following radical prostatectomy (Yang *et al*, 1999). Recent study has also found an association between caveolin-1 expression and less favourable prognoses in oesophageal squamous cell carcinoma (Kato *et al*, 2002). On the other hand, caveolin-1 has been reported as a tumour suppressor gene (Engelman *et al*, 1998b) and its expression level is reduced in several cancers (Lee *et al*, 1998; Racine *et al*, 1999; Bender *et al*, 2000; Wiechen *et al*, 2001a,b). Thus, the role of caveolin-1 in tumour remains controversial.

In the present study, we examined the expression and clinical impact of caveolin-1 in a cohort of patients with pancreatic carcinoma, using immunohistochemical analysis. We found that caveolin-1 is overexpressed in pancreatic carcinoma and has a significant prognostic value for patients with this disease.

## MATERIALS AND METHODS

### Patients and specimens

We studied surgically-resected specimens from 79 patients with pancreatic ductal adenocarcinomas and seven with chronic pancreatitis treated at the Department of Surgical Oncology of Hokkaido University Hospital, the Department of Surgery of Teinekeijinkai Hospital, and the Department of Surgery of Hokkaido Gastroenterology Hospital from 1992 to 1999. Among the 79 patients with cancer, 60 underwent pancreatico-duodenectomy (Whipple operation), 14 underwent distal pancreatecomy and five underwent total pancreatectomy with curative intent. All patients received extended radical lymphadenectomy. Pancreatic resection was not performed in patients with distant site metastases. Any cases of cystadenocarcinoma or mucin-producing carcinoma were excluded from this study. Pancreatic carcinoma tissues were obtained from 44 men and 35 women with a median age of 63 years (range, 31–83 years). The median duration of follow-up was 57.6 months (range, 3.8–103.8 months), and 59 patients (74.7%) died during the follow-up period.

All specimens were fixed in 10% formalin and embedded in paraffin wax. Representative blocks were selected (based primarily on the greatest dimensions of the tumour), and serial 4 μm-thick sections were examined by immunohistochemistry. Histological classification of tumours was based on the World Health Organization criteria. All tumours were staged according to the pTNM pathological classification of the UICC (International Union Against Cancer) (Sobin and Wittekind, 1997). Thirty of 79 patients had positive resection margin for carcinoma, as evaluated microscopically.

### Immunohistochemistry

Immunohistochemical reactions were carried out using the streptavidin–biotin–peroxidase method. Sections were deparaffinized in xylene and rehydrated through a graded series of ethanol/water. Endogenous peroxidase was blocked with 3% hydrogen peroxide for 10 min. Sections were washed twice in phosphate-buffered saline (PBS) and incubated with 10% normal goat serum (Histofine SAB-PO kit; Nichirei, Tokyo, Japan) for 30 min. Primary antibody (anti-caveolin-1 rabbit polyclonal antibody directed against caveolin-1 residues 2–21, Santa Cruz Biotechnology, Santa Cruz, CA, USA) was applied in a 1 : 400 dilution in PBS, and sections were incubated overnight at 4°C. After three additional washes, sections were incubated with polyvalent biotinylated goat anti-rabbit antibody for 30 min at room temperature. Sections were washed three times in PBS and incubated with streptavidin-conjugated peroxidase for 30 min at room temperature. After three additional washes, the reaction product was visualized after incubating with 3,3′-diaminobenzidine tetrahydrochloride (Histofine SAB-PO kit; Nichirei, Tokyo, Japan) for approximately 15 min and then washing with distilled water. Sections were counterstained in haematoxylin for 1 min and mounted in Permount (Micro Slides; Muto-Glass, Tokyo, Japan). The smooth muscle cell segments or endothelium, both known to be abundant in caveolin-1, were used as positive controls. For a negative control, nonimmune purified rabbit serum was used for the primary antibody. The number of stained cells per 1000 was determined under a microscope (Olympus Optical Co, Ltd, Tokyo, Japan) in three visual fields, at a magnification of ×200. When the total number of cancer cells observed under microscope was less than 1000, all cells were counted. When over 50% of all cancer cell cytoplasm was stained, the tumour was considered caveolin-1 positive. This cut-off value (50%) was adopted from previous reports (Yang *et al*, 1999; Kato *et al*, 2002). The present study was performed retrospectively, but all specimens were evaluated by three investigators who were blind to the patients' clinical information.

### Statistical analysis

Caveolin-1 immunoreactivity was assessed for association with clinicopathologic variables using the following statistical tests: the Mann–Whitney test for depth of invasion, pTNM stage and histopathologic grade; the chi-square test or Fisher's exact test for the remaining variables. The Kaplan–Meier method was used to generate survival curves, and survival differences were analysed with the log-rank test, based on the status of caveolin-1 expression. Univariate and multivariate analyses were performed using Cox's proportional hazard regression model. Probability values less than 0.05 were considered statistically significant. All analyses were performed using statistical analysis software (StatView, version 5.0; SAS Institute, Inc. Cary, NC, USA).

## RESULTS

### Caveolin-1 expression of pancreatic carcinoma and chronic pancreatitis specimens

**Figure 1:**
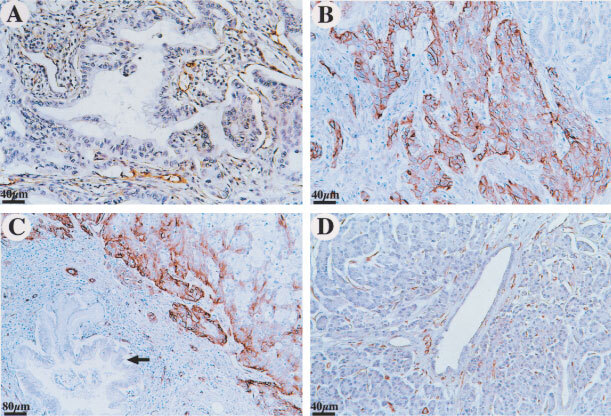
Representative examples of caveolin-1 immunostaining. (**A**) Caveolin-1 immunoreactivity was observed in less than 5% of cancer cells, while endothelial cells showed strong staining as an internal control. (**B**) Strong staining both in the cytoplasm and on the membrane of cancer cells. Over 50% of tumour cells were reactive. (**C**) Non-neoplastic ductal epithelium adjacent to cancer cells showed no immunoreactivity (arrow). (**D**) In chronic pancreatitis, normal ductal epithelium showed no immunoreactivity. (Original magnifications: **A**, **B**, **D**, ×200; **C**, ×100)

Caveolin-1 was expressed both on the cell membrane and in the cytoplasm of cancer cells, as evidenced by the presence of stained granular immunoreaction products. Out of the 79 specimens of pancreatic carcinoma, seventeen specimens were immunoreactive for caveolin-1 in less than 5% of cells (Figure 1A

); 20 specimens were immunoreactive in 5–25% of cells; 10 specimens were immunoreactive in 25–50% of cells; and 32 specimens were immunoreactive in over 50% of cells (Figure 1B). According to our criteria, 32 out of 79 (40.5%) tumours were positive for caveolin-1. Histologically non-neoplastic ductal epithelium adjacent to cancer cells showed little or no immunoreactivity with caveolin-1 (Figure 1C). In all chronic pancreatitis specimens, normal ductal epithelium showed little or no staining, while the smooth muscle and endothelial cells consistently stained positively (Figure 1D).

### Caveolin-1 expression and clinicopathologic variables (Table 1)

**Table 1:**
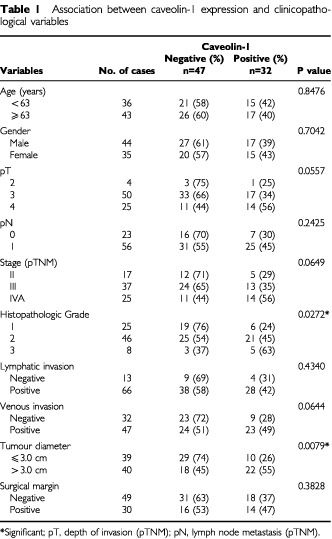
Association between caveolin-1 expression and clinicopatho logical variables

Caveolin-1 expression showed a significant correlation with tumour diameter (*P*=0.0079) and histopathologic grade (*P*=0.0272). No significant association was noted between caveolin-1 expression and other clinicopathologic variables.

### Caveolin-1 expression and prognosis

**Figure 2:**
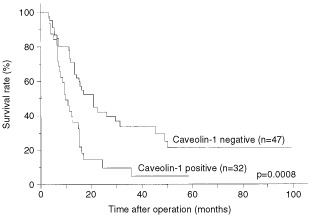
Kaplan–Meier analysis of the overall survival of patients with negative and positive tumour caveolin-1 expression (log-rank test, *P*=0.0008).

Survival curves of patients, grouped according to level of caveolin-1 staining, are shown in Figure 2

. The caveolin-1 positive group had a significantly poorer prognosis than the caveolin-1 negative group (3-year survival rate was 4.8% *vs* 33.8%, respectively) (log-rank test: *P*=0.0008). Upon univariate analysis with Cox's proportional hazards model, lymph node metastasis (*P*=0.0007), tumour diameter (*P*=0.0027), positive surgical margin (*P*=0.0014) and caveolin-1 immunopositivity (*P*=0.0011) were all positively correlated with poor prognosis. Multivariate analyses indicated that caveolin-1 positivity was an independent unfavourable prognostic factor (*P*=0.0358; risk ratio, 1.880; 95% CI, 1.043–3.390), as were the presence of lymph node metastases (*P*=0.0009) and a positive surgical margin (*P*=0.0045) (Table 2

**Table 2:**
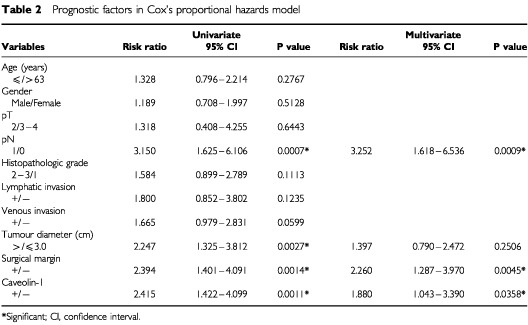
Prognostic factors in Cox's proportional hazards model

).

## DISCUSSION

The present study shows that: (1) the level of caveolin-1 expression is elevated in pancreatic adenocarcinoma relative to non-neoplastic ductal epithelium, (2) caveolin-1 expression is related to tumour diameter and histopathologic grade, and (3) positive caveolin-1 expression is an independent unfavourable prognostic factor following surgical resection.

The expression of caveolin-1 is elevated in various cancer tissues, including prostate cancer, oesophageal squamous cell carcinoma, colon cancer and breast cancer (Yang *et al*, 1998, 1999; Fine *et al*, 2001; Kato *et al*, 2002). In addition, a correlation between caveolin-1 expression and clinicopathological variables has been shown in these cases. In prostate cancer, caveolin-1 expression positively correlated with Gleason score, positive surgical margin and lymph node metastasis (Yang *et al*, 1999). In oesophageal squamous cell carcinoma, expression of caveolin-1 was positively correlated with histopathologic stage, lymph node metastasis and distant metastasis (Kato *et al*, 2002). In the present study, positive caveolin-1 expression was detected in 32 out of 79 tumours (40.5%) in pancreatic carcinoma, while non-neoplastic ductal epithelium showed little or no immunoreactivity. Moreover, caveolin-1 immunopositivity is positively correlated with tumour diameter and histopathologic grade in this cancer as well. These results suggest that caveolin-1 overexpression may contribute to the aggressiveness of pancreatic carcinoma.

Except for the case of prostate cancer, however, the molecular and cellular underpinnings of the relationship between caveolin-1 expression and cancer progression remain unclear. In prostate cancer, caveolin-1 both protects against androgen withdrawal-induced apoptosis *in vitro* and *in vivo* (Nasu *et al*, 1998) and blocks c-*myc*-induced apoptosis in cancer cells (Timme *et al*, 2000). Moreover, caveolin-1 mediates testosterone-stimulated survival/clonal growth and promotes metastatic activity (Li *et al*, 2001), while overexpression of caveolin-1 potentiates ligand-dependent androgen receptor activation (Lu *et al*, 2001). It is well known that prostate cancer is androgen-dependent. Androgen receptor has also been detected in cancerous tissues of pancreatic carcinoma (Corbishley *et al*, 1986), and it is thought that testosterone increases growth of this tumour (Greenway, 1998). Thus, a similar mechanism may be at work in prostate cancer and pancreatic carcinoma.

Interestingly, it has recently been reported that a caveolin-1 mutation at codon 132 was found in human breast cancer specimens and that the mutated caveolin-1 induced cellular transformation, activated the mitogen-activated protein kinase (MAPK)-signalling pathway, and promoted invasion ability in NIH3T3 cells (Hayashi *et al*, 2001). Following up this result, we searched for this caveolin-1 mutation in 11 pancreatic cancer cell lines, but found no mutation in the predicted functional domains (including the scaffolding and membrane-spanning domain) (data not shown). In light of this finding, we suggest that overexpression of wild-type caveolin-1 may be associated with cancer progression in pancreatic carcinoma.

Contrary to the above-mentioned studies, it has been reported that caveolin-1 levels were reduced in a variety of cancer cell lines and cancer specimens (including human breast cancer, lung cancer, colon cancer, ovarian cancer and sarcoma) (Lee *et al*, 1998; Racine *et al*, 1999; Bender *et al*, 2000; Wiechen *et al*, 2001a,b). Under some conditions, caveolin-1 has been shown to suppress growth of specific cell lines *in vitro* and *in vivo* (Koleske *et al*, 1995; Engelman *et al*, 1997; Suzuki *et al*, 1998), and some have suggested that caveolin-1 functions as a tumour suppressor gene (Engelman *et al*, 1998b). The reasons behind this seemingly contradictory evidence remain unclear. Lee *et al* (2000) suggest that the diverse effects of caveolin-1 may be mediated by different regions of the caveolin-1 molecule, and may depend on the expression levels of other coexpressed molecules. It has been reported that the oncosuppressive effect of caveolin-1 is mediated through the caveolin-1 scaffolding domain (residues 82–101) (Okamoto *et al*, 1998). c-Src, however, induces phosphorylation of caveolin-1 at residue tyrosine 14. Tyrosine 14-phosphorylated caveolin-1 confers binding to growth factor receptor-binding protein 7 (Grb7) and augments both anchorage-independent growth and epidermal growth factor (EGF)-stimulated cell migration (Lee *et al*, 2000). In pancreatic carcinoma, Src kinase overexpression and activation has been reported (Lutz *et al*, 1998). Thus, caveolin-1 might cooperate with other molecules, such as c-Src and Grb7, to stimulate tumour growth in pancreatic carcinoma.

This is the first study demonstrating the prognostic significance of caveolin-1 expression in pancreatic carcinoma. The 3-year survival rate following surgical resection of the caveolin-1 negative group was 33.8%, while that in the caveolin-1 positive group was only 4.8%. Furthermore, multivariate analysis demonstrated that positive caveolin-1 expression is an independent negative prognostic factor. These results can increase the accuracy of prognosis for patients with pancreatic carcinoma, following surgical resection. Caveolin-1 overexpression in resected specimens may be a useful index of adjuvant therapy for the patients with a high risk of poor prognosis.

In summary, overexpression of caveolin-1 in pancreatic carcinoma may contribute to tumour progression and be a negative prognostic predictor following surgery. For patients with a tumour overexpressing caveolin-1, closer follow-up should be performed to find recurrence, and adjuvant therapy may be beneficial. However, at present, the role of caveolin-1 in pancreatic carcinoma remains unclear, and elucidation awaits further investigation.
